# Employee perceptions of non-communicable diseases health risks, absenteeism and the role of organisational support in a South African pharmaceutical manufacturing company

**DOI:** 10.1371/journal.pone.0279008

**Published:** 2022-12-12

**Authors:** Philippe Jean-Luc Gradidge, Daleen Casteleijn, António Palmeira, Ralph Maddison, Catherine E. Draper

**Affiliations:** 1 Department of Exercise Science and Sports Medicine, School of Therapeutic Sciences, Faculty of Health Sciences, University of the Witwatersrand, Johannesburg, South Africa; 2 Occupational Therapy Department, School of Therapeutic Sciences, Faculty of Health Sciences, University of the Witwatersrand, Johannesburg, South Africa; 3 CIDEFES, University Lusóphone, Lisbon, Portugal; 4 Executive Director, International Society of Behavioral Nutrition and Physical Activity (ISBNPA), Lisbon, Portugal; 5 Institute for Physical Activity and Nutrition, Deakin University, Geelong, Victoria, Australia; 6 SAMRC/Wits Developmental Pathways for Health Research Unit, School of Clinical Medicine, Faculty of Health Sciences, University of the Witwatersrand, Johannesburg, South Africa; Onom Foundation, MONGOLIA

## Abstract

**Background:**

The growing prevalence of non-communicable diseases (NCDs) in South African workers has been shown to be associated with absenteeism and increased risk of morbidity. Low-income workers living in urban settings are particularly vulnerable. Consultation with workers is crucial for understanding risks and identifying opportunities for intervention to promote health in the workplace. The purpose of this study was to examine South African pharmaceutical manufacturing workers’ perspectives of health risk factors and absenteeism, and to identify how they perceived a role for the organisation to initiate interventions to improve their health.

**Materials and methods:**

Five focus groups were conducted to capture 27 employees’ perspectives. The semi-structured focus group discussions were recorded and analysed using a thematic content analysis approach.

**Results:**

Participants indicated that they were aware of behavioural health risks such as prolonged sitting. They showed insight into strategies to prevent injuries and stay healthy, but also expressed dissatisfaction about the lack of organisational support, leading to stress and consequently absenteeism. Participants emphasized the responsibility of the organisation to support a range of health promoting strategies

**Conclusions:**

The findings of this study are important for cultivating a tailored workplace intervention to reduce NCD risk factors in the pharmaceutical manufacturing workforce. It is vital that these be supported by leadership of the company through the provision of funding and the development of internal healthcare services.

## Background

Chronic non-communicable diseases (NCDs) remain the leading causes of premature mortality amongst both sexes in low- and middle-income countries (LMICs) [1]. Working-age populations in these settings are confronted with a high risk of living and working with chronic conditions. These are mostly driven by the increasing presence of obesity and diabetes in the region [2, 3]. Although these conditions are preventable, effective public health initiatives are limited and public healthcare systems are frequently under resourced and in crisis in African countries [4]. Workers employed in the South African manufacturing sector are particularly vulnerable to comorbidities and high absenteeism. This is due to historic socioeconomic disparities and limited access to available heath systems [5–7].

Absenteeism refers to absences from scheduled work due to illness [8]. This is a major concern for company economic growth due to production days lost. Cross-sectional and longitudinal studies indicate that absenteeism is highest in participants with existing chronic conditions and workers at risk of chronic diseases due to obesity-related behaviours [9, 10]. Recent evidence indicates a high prevalence of NCDs and other chronic conditions in South African workers and across various industries due to insufficient physical activity and the increasing obesity pandemic in the country [7, 11, 12]. Systematic reviews of behavioural workplace interventions to address these risks stress the need for further investigation on the management of risk factors for chronic conditions for employee health [13, 14], and the sub-sequent reduction in absenteeism [15]. Quality data on South African workplace interventions are particularly limited, however existing evidence shows promise for improving the cardiometabolic health of workers [16, 17].

The first of only two known South African studies, a follow-up study of power plant workers in the Western Cape region of South Africa, demonstrated significant improvements in cardiovascular and mental health profiles following a tailored workplace behavioural intervention [17]. The participants in this study were offered healthier meal options at a reduced cost through on-site food vendors, provided with opportunities for physical activity and delivered health education through wellness drives in the company. The intervention was beneficial for South African workers because of the confirmed acceptance from the participants and management of the company, and specific to the setting. The second randomised controlled trial, a study conducted in employees of a rural South African higher learning institution, demonstrated improvements in cardiometabolic health profile following a programme of structured workplace walking [16]. These data confirm the benefits of workplace interventions for reducing NCD risk factors in the South African workforce. Findings of a South African qualitative study recommends a robust understanding of the contextual determinants of disease in the workplace and the development of tailored strategies requires consultation with employees as it is important to collect data on factors that impact implementation [18].

However, the evidence showing how to implement and use these interventions, or what employees think of these interventions is lacking. Qualitative research can provide the necessary formative work for a hybrid-effectiveness study design [19] to understand how to develop and implement workplace interventions, that incorporate employees’ views, to address the burden of NCDs and reduce absenteeism.. Therefore, the aim of this study with South African pharmaceutical manufacturing workers was to qualitatively examine workers’ perceptions of health risks and absenteeism, and to determine how workers viewed a role for management in addressing these factors.

## Materials and methods

### Study population

The study was conducted at a pharmaceutical manufacturing company in Johannesburg, South Africa. The company operates 24 hours/day, 7 days/week. The types of jobs in this company were graded using the Paterson grade system [20]. A representation of workers from all grades were invited to participate in the focus groups from 145 participants (grades A: 28 (19.3%), B: 86 (59.3%), C: 26 (17.9%), D: 4 (2.8%) and E: 1 (0.7%). The groups were a mix of employees to elicit a wide range of information and perceptions from the group. For this study, purposive sampling was used with inclusion criteria of participants being above 18 years old, employed more than one year at the company, shift work (including morning (conventional daylight hours), afternoon, overnight, or swing), and willing to participate in a focus group. The study was approved by the Human Research Ethics Committee (Medical), University of the Witwatersrand (M190224). All participants gave written informed consent for their participation.

### Data collection

A series of eight focus groups, each with six to eight participants were planned. Focus groups were conducted using a semi-structured schedule to facilitate a variety of point of views from June 2019 to March 2020. Guide questions were modified from a previous South African that explored the perceptions of employees towards workplace interventions [18] to explore participants’ perceptions of physical and psychological health risks and absenteeism in the workplace and, the role of the company to support employee-driven strategies to reduce these concerns (S1 File). During the focus group discussions, questions about sick leave utilisation, physical activity, mental health, and the role of the organisation to support strategies at work. The semi-structured nature of the focus groups allowed participants to raise issues of relevance to them, outside of the questions posed to the group by the facilitator. The facilitator had to be aware of power dynamics between focus groups participants as the hierarchy of positions might have influenced the information that came forward.

A moderator, fluent in English and other main spoken South African languages of the region (IsiZulu, seSotho, Tshivenda), familiar with the data and experienced in qualitative research led the focus groups. This person was responsible for facilitation of the discussion. A co-moderator was responsible for note taking during these focus group discussions. The focus groups continued until the researchers reached consensus on data saturation. Saturation was reached after five focus groups. All focus groups were recorded using a Philips DVT2510 audio recorder with verbal consent of the participants on a portable recorder. The participants were asked to provide simple demographic characteristics including age, type of work, level of employment, and duration at the company.

### Data analysis

First, the recordings were transcribed verbatim by an independent transcriber who also anonymised participants. A reflexive and adaptable thematic analysis approach [21] for coding and theme development was carried out using a combination of inductive and deductive approaches. The questions in the focus group guide were used to develop the initial coding framework; inductive open coding was used in cases where the codes could be derived from the data. For this inductive open coding, the researchers (DC and PJG) came up with their own names for themes, subthemes, and codes. Five transcribed documents were analysed using Atlas.ti 9 (ATLAS.ti Scientific Software Development GmbH), applying the revised coding framework. Codes were applied to the transcripts while the text was read line-by-line and interpreted. Two data coders (DC and PJG) independently coded the data and identified themes and subthemes. Differences and uncertainties were discussed and resolved between the coders (DC and PJG) and CED reviewed these interpretations.

## Results

A total of 27 participants participated in the focus groups and provided data (Table 1); the majority were female (16, 59.3%), in the ‘B’ Paterson grade, and aged between 23 and 59 years of age. Mean length of employment was 5.8 ± 4.8 years. Most participants had not completed high school (22, 81.5%) and 10 (27%) were conventional morning shift workers. Three themes were identified including: (1) physical and psychological health risks, (2) absenteeism in the workplace and (3) organisational support for employee health. These themes captured the perspectives of workers on health risks in the workplace and absenteeism and illustrative quotes are presented.

**Table 1:** Demographic characteristics of participants (n = 27).

Number and sample size of groups	Females n (%)	Average age (years)	Average duration employed (years)	Paterson job grade n (%)	Example job titles	Type of shift work n (%)
Focus group 1	4 (14.8)	42.6	8.5	A: 2 (7.4)	Production, operator, IT technician, Buyer, Creditors clerk, Site service officer, Microbiologist, Pharmacist assistant.	Morning: 3 (11.1)
				B: 5 (18.5)		
(n = 7)						Afternoon: 4 (14.8)
Focus group 2	4 (14.8)	30.2	3.7	A: 1 (3.7)	Security, Formulator, Junior planner, Machine operator, HR officer	Morning: 1 (3.7)
(n = 5)						
				B: 3 (11.1)		Afternoon: 4 (14.8)
				C: 1 (3.7)		
Focus group 3	0 (0)	36.3	8.3	A: 1 (3.7)	Quality assurance technician, Production operator, Stock controller, Tablet making supervisor	Morning:1 (3.7)
				B: 3 (11.1)		
						Afternoon: 3 (11.1)
(n = 4)						
Focus group 4	5 (18.5)	32.3	4.3	B: 3 (11.1)	Documentation clerk, Production operators, Cleaner, Machine operator	Morning: 4 (14.8)
(n = 6)				C: 3 (11.1)		
						Afternoon: 2 (7.4)
Focus group 5	3 (11.1)	41	4.2	A: 1 (3.7)	Microbiologist, Pharmacist assistant, Accounts officer, Production operator, Quality control analyst.	Morning: 1 (3.7)
				B: 4 (14.8)		
(n = 5)						
						Afternoon: 4 (14.8)

### Physical and psychological health risks

The relationship between occupation demands and environmental factors were demonstrated in how workers in the study discussed about behaviours that exemplify health risks, as illustrated in Table 2.

**Table 2:** Physical and psychological health, sub-themes and illustrative quotes.

***a) Barriers to physical activity***	“When I feel like I’ve got pains in my back, I just walk–just walk.” (D5/06.19/I8)
	“I can lift things, but I have to take care of my back.” (D5/06.19/I2)
	“Cooking, checking the books, checking the homeworks [school work with participant’s children]. After that you have to see if the house is tidy or what, washing the dishes and the kids as well.” (Gr4/03.20/I1)
	“I think little bit of exercise is needed but we wake up early in the morning, we knock off late in the afternoon, so there is no time for us to do any exercise.” (Gr4/03.20/I1)
***b) Movement during working hours***	“I am working as a cleaner so I am just keeping on walking.” (Gr5/03.20/I2)
	“You know maybe that is why most of us we are not so healthy because we tend to think that because we do a lot of walking, there is no need for us to exercise.” (Gr3/07.19/I2)
	“Also increases our chances of back damage because you are constantly trying to adjust yourself to keep yourself up straight.” (Gr5/03.20/I2)
***c) Psychological job demands***	“It is the pressure here, when you knock off it is fine, it is knock off time, but when you go home, you also think about that because oh it was too much pressure.” (Gr3/07.19/I4)
	“I think it comes in waves. You are not depressed all the time. It’s like when circumstance hit you that is when you react to what you perceive.” (Gr5/03.20/I1)
***d) Aggravating factors for physical and psychological health***	“The rotating shifts yes. I think those, that one is the problem… your body cannot adjust quickly to the, to the time zones, to the time shifts.” (Gr3/07.19/I3)
	“I think they need to, to show us the appreciation, maybe the end of the month when we reach the target, maybe they need to give us maybe some vouchers, they don’t do that.” (Gr5/03.20/I3)

#### Barriers to physical activity

Many participants commented that their work was physically demanding, with people experiencing low back pain. They also reported that they were very physically tired by the time they reached home after work. The participants reported that they resorted to sedentary activities such as watching television. Some participants, however, did not view the back pain as a barrier to physical activity at home. These participants chose to walk everyday as a conservative form of pain management. While participants acknowledged the benefits associated with exercise, some participants reported limited opportunities during work hours due to time constraints with usual expectations of the job. Others reported scheduling time after hours for more intense exercise sessions. These structured exercise regimens included running and resistance training at their local fitness centres.

#### Movement during working hours

The working environment was identified as a setting for occupation-related physical activity by most participants. Participants expressed that they were sufficiently physically active during vocational hours as part of their jobs. For example, participants were expected to participate in intermittent walking, standing and stair-climbing as the job required. This viewpoint was not shared by all participants as some noted that they did not engage in enough physical activity at work.

Some participants were worried about the health implications of sedentary activities during occupation hours. These included the harmful effects on physical health due to extended sitting time during production and sorting work, seating that does not provide ergonomic support for the lower back and sitting during planned lunch breaks. Participants suggested that those concerns could be addressed by rotating job roles between sitting and standing activities and replacing the current chairs with adjustable ergonomic chairs.

#### Psychological job demands

Most of the participants expressed that they were not capable of dealing with work stressors, such as increased consumer demand for products. This had a negative effect on their psychological health. They described the workplace as a pressured environment and agreed that they felt constantly stressed, even at home after a workday. However, one participant commented that despite poor working conditions, they were grateful for being employed. Other participants felt that the stressors at work fluctuated depending on the demand of the market and observed that healthy coping strategies to reduce personal stress were limited. The participants perceived that increased physical activity was associated with improved physical and mental health, reduced reliance on medication for chronic conditions and therefore lower financial stress.

#### Aggravating factors for physical and psychological health

Participants who were non-conventional morning shift workers (10, 37%) commented that their sleep patterns were disrupted and felt that this was the reason for their lack of energy. Others mentioned that verbal feedback and approval from company management was an important cue of their personal work performance. For instance, participants felt that there was a lack of recognition and appreciation for meeting company targets. They believed this was the reason for a general dissatisfaction with the company and a contributor to personal stress. Participants commented that the monthly salary was lower than the salaries in other manufacturing industries.

### Absenteeism in the workplace

This theme captures the reasons for absenteeism at the company in which the participants are employed, and illustrative quotes are displayed in Table 3.

**Table 3:** Absenteeism in the workplace, sub-theme and illustrative quotes.

***Patterns and reasons for absenteeism***	“I think women because they have to take care of the kids.” (Gr5/03.20/I3)
	“It’s the guys, you. You all know it’s the guys; They are always sick, especially on Monday.” (Gr3/07.19/I1)
	“I also think the same because the kind of the job that the men are doing, they are more likely to do a heavier job than the ladies.” (Gr3/07.19/I1)
	“They (females) go for monthly check-ups and they get dates by the clinic or a hospital to say you must come.” (Gr5/03.20/I1)
	“Another thing is, even if your husband is sick, you have to accompany them sometimes, depending on how sick they are. Also the kids as well. But with men obviously I don’t think there is a lot of men who take their babies to the clinic. It’s always that, you know. It’s the norm that the mother is supposed to take them.” (Gr1/06.19/I3)

#### Patterns and reasons for absenteeism in the company

Most participants reported understanding the difference between absenteeism and vacation leave. Males expressed that female employees were expected to oversee the family responsibilities. Domestic tasks such as taking their children to the local clinic for scheduled appointments were perceived as requiring additional leave. Indeed, many participants mentioned domestic responsibilities as a key reason for absenteeism. Participants commented that the company policy and government labour regulations allowed them to access the family responsibility leave plan as the need arose.

One male participant expressed concern for females having to take sick leave for personal monthly medical assessments and family planning. Most female participants, however, reported that some female employees were single parents with additional social and financial challenges. Female participants viewed that male workers abused the absenteeism policy because they used it to recover from weekend binge drinking. Similarly, female participants expressed a concern about males in the company taking sick leave for minor ailments such as aches and pains. Male participants agreed, describing that the reason for this was due to fatigue from physical labour at home.

### Organisational support for employee health

When asked if the company could support workplace interventions in the organisation, participants remarked that the leadership of the organisation could contribute to introducing health services at work, health education, and activities that promote social interactions among staff during physical exercise in the workplace. Illustrative quotes are summarised in Table 4.

**Table 4:** Organisational support for employee health, sub-themes and illustrative quotes.

***a) Heath service expectations***	“It’s a bed, it’s a wheelchair, first aid kit, stretcher if somebody is sick.” (Gr1/06.19/I5)
	“If you feel funny, just go for a check-up, see isn’t your blood pressure too high or whatever.” (Gr1/06.19/I7)
	“We think it will be much better if … maybe the company could contribute half or 50% of it, because some of us don’t earn enough to actually pay for that medical aid.” (D4/06.20/I1)
	“I think they also need to provide counselling” (Gr2/06.19/I3)
	“The last fainting incident we had was because, I don’t know, probably the person was stressed.” (Gr2/06.19/I1)
***b) Health education services***	“They don’t know, people don’t know that even though you have got hypertension, there might be high cholesterol also developing, and glucose. To live healthy, someone to educate, and someone who the people can believe.” (Gr3/07.19/I2)
***c) Social and physical activities***	“During winter, when you arrive, it’s almost half past 6 [18:30], its dark. During rain time, it’s raining, you cannot do anything. But I will suggest that maybe [the company] could provide us with a little bit of gym so that we can exercise maybe after hours, let’s just exercise just to keep our wellbeing.” (Gr4/03.20/I1)
	“Like a fun day thing and like a body building facility to re-connect with the employers and managers, like a team building exercise.” (Gr1/06.19/I2)

#### Suggestions for health services

Participants were asked to discuss what health services were required in the company. There was a general view that a professional registered nurse was required onsite to cover the overall occupational health services in the factory, particularly as private medical aid was unaffordable with low monthly incomes. Participants mentioned primary prevention and medical screening services as the motivator for addressing health risks in the workplace. Some participants recalled a serious injury on duty incident where an employee lost a finger while operating machinery on the production line. One participant reported a fellow colleague having an epileptic seizure during work and added that this incident could have had fatal consequences. A major concern was that the company did not have trained first aiders on duty to respond to these incidences and the participants were not aware of the company having a first aid kit. Many felt strongly about having a dedicated room for first aid equipment that would be administered by a professional nurse to serve the health requirements of the staff. The participants agreed that this situation and other work-related stressors made them anxious and suggested management providing counselling services as a coping strategy.

#### Suggestions for health education

Most of the participants expressed concern about their personal health and viewed their lifestyles as contributing to the increased risk of disease. However, they observed that it was difficult to maintain a healthy lifestyle and required more information on health behaviours. Participants thought that the information should be provided by qualified healthcare providers. The diseases mentioned as most concerning were hypertension, high cholesterol, and type 2 diabetes.

#### Suggestions for social and physical activities

Both male and female participants discussed the potential of a fitness facility to exercise during and after work hours before commuting home. While participants reported on the benefits of an onsite facility, numerous participants were also mindful of the financial implications and felt that an outdoor gym on the company property could serve a similar purpose but cost less.

Another facilitator of physical activity noted by participants was the introduction of recreational activities at the company such as football, netball and cricket. Many participants mentioned that these activities were important to support social cohesion and collegiality amongst employees. In addition, multiple participants reported that they were in support of introducing activities, games and team-building sessions at the workplace. One participant suggested having monthly dietary weight loss competitions with monetary incentives. There was agreement among study participants that these home-grown initiatives are important for employee health and thus emphasised the need for support from company management.

## Discussion

This qualitative study was conducted to explore pharmaceutical manufacturing workers’ perceptions of physical and psychological health risks and absenteeism, and to understand their perceptions of management’s roles and responsibilities in lowering these risk factors, since data are limited in a South African setting. The findings of our study lay the foundation to develop and implement a workplace intervention that is informed by employees’ views.

Similar to previous studies [22], participants believed that they experienced excess fatigue after working a full day at the company. They felt that they were limited in their knowledge of exercise modalities and viewed the feeling of tiredness as a barrier to healthy behaviour at work. Despite many barriers to physical health being discussed, participants realised the benefits of physical movement, in various domains of physical activity as observed in other studies [23]. Physical activity at home was perceived as positive and conducive to one’s health but participants believed they lacked time to pursue exercise. This perception was also noted in similar research but specifically with female employees in cleaning services roles [24]. These findings highlight the potential for interventions aiming, for example, at facilitating the scheduling of opportunities to engage in activities in various domains of physical activity. In the case of developing a workplace intervention, we suggest using a theoretical framework, such as the COM-B (Capability, Opportunity, Motivation—Behavior) [25]. The COM-B model was used to identify the themes from the focus groups have the intervention will seek to address the physical and psychological (Capability), social and physical (Opportunity), and automatic and reflective (Motivation) elements and determine its effect on Behavior change in this cohort of pharmaceutical workers (Table 5).

**Table 5:** Mapping of the COM-B model to the themes [25].

COM-B component	Related theme
Capability	Psychological job demands
Opportunity	Barriers to physical activity
	Movement during working hours
	Barrier
	Health education services
	Social and physical activities
Motivation	Aggravating factors for physical and psychological health
	Patterns and reasons for absenteeism
	Health service expectations

Occupation-related physical activities, such as stair-climbing during work hours, were discussed as positive opportunities amongst study participants. However, the nature of the job role needs to be considered as study participants discussed some roles in pharmaceutical production required employees to remain seated. Previous literature suggests that interrupting prolonged sitting with standing and walking is associated with improved cardiometabolic outcomes, while extend sitting during work increases the risk of cardiovascular diseases [26]. Some examples that could be introduced in this setting include height-adjusted standing desks and prompts to reduce sitting as observed in previous studies [27, 28]. In fact, previous evidence has shown the medication of work environment with these measures can also reduce the presence of chronic low back pain [29] and subsequent absenteeism especially in physically and cognitively demanding roles [30]. These interventions would be focusing on the Opportunity component of the COM-B model.

Participants discussed the psychological job demands and other aggravating health risk factors that resulted in dissatisfaction with the working environment. A few coping strategies were proposed in the focus groups, which indicated the readiness of staff to address work stressors and specifically by department. Previous studies have shown that the consequences of this negative perception includes a mistrust of management and general apathy amongst staff [24]. It is therefore important to identify and understand the reasons for experiencing dissatisfaction at work, particularly as these concerns could be addressed through open dialogue to develop workplace interventions. A study conducted at a telecommunications conducted in Malaysia [31], for instance, showed an increase in job satisfaction, decrease in stress and absenteeism after implementing an employee workplace intervention. These results can be linked to COM-B’s Motivation component; the way the social context, including job pressures, are known to affect one’s own motivation [32].

Participants were concerned about absenteeism in the company, but there were mixed views of how this concern was influenced by gender. Our study findings show that males perceived females having higher absenteeism due to family responsibilities and childcare, which is similar to earlier studies comparing reasons for absenteeism by gender [33, 34]. A study in India on absenteeism in pharmaceutical industrial workers revealed that 30% of sick leave was due to social responsibilities and family health concerns [35]. The male participants in our study perceived that female employees had a social duty to address the health concerns of themselves and their families. On the other hand, female participants understood that male workers were absent due to excess fatigue and binge drinking on weekends. This perception was opposed by the male participants, however a study from New Zealand reported that high absenteeism was predicted by being male, <25 years, consuming a high amount of alcohol and employed in a stressful job role [36].

Participants reflected on the importance of management to support health in the company. These included attending to injuries on duty and monitoring and prevention and monitoring of NCDs. The nurse could also act as a provider of basic health education, giving evidence-based protocol on how to manage chronic conditions by modifying lifestyle. The participants suggested having counselling sessions with a recognised counsellor to assist with work stress and discussed to the concept of affordable medical aid coverage for staff. These recommendations were viewed as management’s responsibility to improve the health status of employees. Although, the capital to set up these recommendations are relatively high, most of the participants’ suggestions for workplace interventions were reasonably low-cost such as the introduction of regular social physical activities at the company to incentivise health living. The study conducted at a South African power plant has confirmed that workplace interventions lower NCD risks among staff [17], however these efforts require tailoring according to context and employee needs [37]. The study participants understood their own role in addressing personal health concerns and management’s responsibility for facilitating improved health amongst staff.

This study did not consider the views of the company management as this would have limited open discussion of subordinates, and future research should aim to comprehend whether these views concur with those of the employees, and how these may influence the development of lifestyle interventions in the workplace. These study findings may therefore not be pertinent to other work settings as this investigation was carried out in one pharmaceutical company.

## Conclusions

This study highlighted that pharmaceutical employees understand the importance of executing behaviour modification at work to offset the potential adverse effects of disease and absenteeism. There were appeals for prioritising primary healthcare services in the company since private medical aid was unaffordable, but also as a mechanism to manage chronic diseases and medical emergencies. Integrating the support of the organisation into these strategies can help maintain occupation-related physical activity, lower absenteeism and promote improved cardiovascular health amongst employees.

## Supporting information

S1 File Interview guide for focus group discussions. (PDF)
