# Supplementary material for: Employee perceptions of non-communicable diseases health risks, absenteeism and the role of organisational support in a South African pharmaceutical manufacturing company
Source: PLoS One. 2022 Dec 12;17(12):e0279008. doi: 10.1371/journal.pone.0279008 (PMC9744274; doi:10.1371/journal.pone.0279008)
Supplement: S1 File — (PDF) [file pone.0279008.s001.pdf]

S1 Interview guide for focus group discussions

Employee perceptions of non-communicable diseases health risks, absenteeism and the role of organisational support in a South African pharmaceutical manufacturing company

Physical and psychological health risks

Barriers to physical activity

- What, in your opinion, are barriers to physical activity at work?
  - What chronic diseases do you think will increase if you do not change anything about this?
  - What are some health benefits to overcoming the barriers to activity?
  - What would it take for you to increase the importance of physical activity and exercise?

Physical activity during vocational time

- Do you think that workers can be physically active at work?
- Do you think that some job roles are more active than others?
  - How are these jobs considered as more physically demanding?
- What strategies could be used to increase physical activity for employees during work?
- What is your opinion on increasing physical activity during commuting to work?

Mental health

- Are you happy at work?
- What is your opinion about depression and stress amongst staff?
- In your opinion, are you coping with work stress and depression?
- What is your opinion about the causes of work stress and depression?
- What strategies could be used to reduce work stress and depression?

Absenteeism in the workplace

- What in your opinion, is considered as absenteeism?
- In your opinion, are the staff taking much sick leave?
- Do you feel that men or women are taking more sick leave?

    - What do you think about this?
- What in your opinion would be the reasons for men and, for women taking sick leave?
    - What do you propose to do about this?

Organisational support for employee health

- What are some ways to address or overcome the health risks in the workplace?
    - Are there behavioural strategies that can be introduced at work?
- Do you think that the company should have a role in promoting healthy behaviour during working hours?
    - What role should management having in increasing physical activity at work?
    - What do you think about this?
- What in your opinion do you consider as priority healthcare services in the company?
    - What can be done to improve these services?
    - In your opinion, should these offerings include specific education on chronic diseases? What do you think about this?

Thank you for participating in the focus group discussions.
